# Bayesian Mixed Models Approach to Exploring Resilience: Impact of Stress on Subjective Health and Affects Over Time During the COVID‐19 Pandemic

**DOI:** 10.1002/mpr.70050

**Published:** 2026-01-28

**Authors:** Markus Schepers, Irene Schmidtmann, Sarah K. Schäfer, Simge Yilmaz, Rieke Baumkötter, Alica Hartmann, Julia Petersen, Nora Hettich‐Damm, Philipp Wild, Daniela Zahn, Daniel Wollschläger

**Affiliations:** ^1^ Institute of Medical Biostatistics Epidemiology and Informatics University Medical Center of the Johannes Gutenberg‐University Mainz Mainz Germany; ^2^ Leibniz Institute for Resilience Research Mainz Germany; ^3^ Clinical Psychology Psychotherapy & Diagnostics Technische Universität Braunschweig Braunschweig Germany; ^4^ Preventive Cardiology and Medical Prevention Center of Cardiology University Medical Center of the Johannes Gutenberg‐University Mainz Mainz Germany; ^5^ German Center for Cardiovascular Research (DZHK) Partner Site Rhine Main University Medical Center of the Johannes Gutenberg University Mainz Mainz Germany; ^6^ Department of Ophthalmology University Medical Center of the Johannes Gutenberg University Mainz Mainz Germany; ^7^ Department of Psychosomatic Medicine and Psychotherapy University Medical Center of the Johannes Gutenberg‐University Mainz Mainz Germany; ^8^ Center for Thrombosis and Hemostasis (CTH) University Medical Center of the Johannes Gutenberg University Mainz Mainz Germany; ^9^ Institute of Molecular Biology (IMB) Mainz Germany; ^10^ Health Sciences Hochschule Fulda University of Applied Sciences Fulda Germany

## Abstract

**Background:**

Profound stressors such as the COVID‐19 pandemic have highlighted the importance of understanding resilience mechanisms and approaches for quantifying them in longitudinal studies.

**Methods:**

We used Bayesian mixed models to analyze resilience dynamics with ordinal dependent variables: subjective physical and mental health, and fear, sadness, and anger. The models included fixed effects for individual stressors and random intercepts for participants, applied to the Gutenberg‐COVID‐19 cohort study.

**Results:**

There were 206,912 responses from 7386 participants (mean age 55.09 years, 51.52% women) over one year (Oct 29, 2020 ‐ Oct 25, 2021). Social stressors, such as loss of social contacts, had stronger negative associations with health and negative affects than work‐related stress. Subjective health and emotions declined during lockdowns but quickly recovered afterward.

**Conclusion:**

Our longitudinal study design and mixed‐model analysis highlight the role of social stress and encourage further research into protective factors like social support and positive reappraisal.

## 1 Introduction

Apart from the immediate disease and death toll, the COVID‐19 pandemic caused severe psychological stress and already early on, triggered calls for more research on mental health and psychological resilience (Holmes et al. 2020). As psychological resilience and its importance is easy to grasp intuitively, yet difficult to define precisely, multiple conceptualizations have been developed. In particular, resilience has been described as a capacity, a mechanism or an outcome (Arnold 2023). Most generally, resilience is the ability (process or outcome) of coping well with stress. According to the currently most popular definition, stress resilience is the phenomenon that some people maintain their mental health despite exposure to adversity or show only temporary impairments followed by quick recovery (Kalisch et al. 2024). There is a long history of stress theory, approaching it as a stress reaction, as a transactional process or as a stimulus. Stress as a stress reaction focuses on the body's physiological and psychological responses to stressors. Stress as a transactional process views stress as a dynamic interaction between the individual and their environment, emphasizing cognitive appraisals and coping mechanisms. Stress as a stimulus defines stress in terms of external stressors or stimuli that cause stress reactions in individuals. For instance, according to Hans Selye, stress is the non‐specific response of the body to any demand placed upon it (Selye 1936, 1950; APA 2024), or, according to Bruce McEwen and Jaap Koolhaas, the conditions where an environmental demand exceeds the natural regulatory capacity of an organism (Koolhaas et al. 2011). The stress is caused by a stressor, which is any event or environment that individuals might consider demanding, challenging, and/or threatening to individual safety. The stressors we consider here are changes in daily life and the pandemic overall (see method's section).

The conceptualization of resilience as a time‐dependent process in terms of recovery from (or lack of recovery from) experience of stressors requires the analysis of longitudinal data, even when we are only interested in a single (static and time‐independent) estimate of resilience. Recently, it has been proposed to measure resilience cross‐sectionally as residuals in a linear regression with the dependent variable mental health issues and explanatory variables such as the number of stressors currently experienced (Kalisch et al. 2021). Inspired by that work, we here propose to measure resilience using ordinal mixed models, thereby extending the previous method in at least two ways to the longitudinal data setting: firstly, the random intercepts of the mixed models yield estimates with confidence (or credible) intervals that are separate from the irreducible statistical error. Secondly, the mixed models take into account that measurements within the same participant are dependent (i.e., more likely to cluster around one value). Furthermore, as psychological measurements based on surveys are often on an ordinal scale (at least for our data), we use ordinal logistic modeling. In this framework, estimated odds ratios represent the relative odds of being in a higher versus lower category of the outcome per unit increase in a predictor. If the values of the dependent variable are ordered such that higher values are better, then ORs less than 1 indicate that higher stress is associated with worse outcomes, while ORs greater than 1 indicate the opposite.

We apply this analysis framework to the Gutenberg‐COVID‐19 study, which is a large cohort study with more than 7000 participants with around 30 time points each, and propose this analysis framework for future longitudinal studies of resilience.

## 2 Methods

### 2.1 Data

We analyzed longitudinal app survey data from the Gutenberg COVID‐19 Study (GCS). The GCS was initiated after the outbreak of SARS‐CoV‐2, by re‐inviting participants from the Gutenberg Health Study (Wild 2012), a large, population‐based prospective single‐center cohort study started in 2007 with more than 15,000 participants, along with newly recruited younger participants. The participants were sampled in the target area of Mainz‐Bingen, stratified by age, sex and place of residence. Around 80% of GCS‐participants were recruited from the GHS, the remainder were newly included younger participants (aged between 25 and 44 years). At the GCS study center, participants underwent a computer‐assisted personal interview and sampling of biomaterial (at the start of the study, i.e. during the pandemic). In preparation for their appointments, study participants were sent questionnaires in advance. Additionally, all GCS‐participants were invited to take part in an app survey conducted weekly, or in case of a positive SARS‐CoV‐2 test result, every 2 days (where around 75% of GCS participants ended up taking part in the app survey). An app report consisted of six blocks of questions and took 10–15 min to complete.

The baseline examination of the GCS took place from October 2020 to April 2021 (T1). The first GCS follow‐up was conducted from March 2021 to June 2021 (T2). The app survey took place from October 29, 2020 to October 25, 2021.

The full GCS sample consists of 10,250 participants (age 56.1 ± 15.7 years, 50.8% female), but we focus on the 7386 participants (age 55.1 ± 13.6 years, 51.5% female) who also took part in the app survey as we are interested in a more fine‐grained temporal resolution.

The principles of Good Clinical Practice (GCP), Good Epidemiological Practice (GEP), and the ethical guidelines set forth in the Declaration of Helsinki were integrated throughout the entire process of the study design, execution, and analysis. Additionally, we adhered to the requirements outlined in the Federal Data Protection Act. The Ethics Committee of the Rhineland‐Palatinate Medical Association and the Data Protection Officer of the Johannes Gutenberg‐University Medical Center Mainz thoroughly assessed all pertinent documentation for both the Gutenberg Health Study and the Gutenberg COVID‐19 Study, granting their unequivocal approval. Furthermore, the data protection commissioner of Rhineland‐Palatinate approved the selection of the sample through citizens' registration offices.

### 2.2 Variables of Interest

For the description of the study sample we consider sociodemographic variables such as age, sex, employment status as well as recent COVID‐tests and contacts with potential or confirmed cases of COVID‐19.

For the mixed modeling of resilience in separate models (per dependent variable), the dependent variables are subjective physical and mental health as well as fear, sadness and anger, respectively. Physical and mental health were measured on a scale from one to four, where 1 is best (and 4 is worst), while fear, sadness and anger were measured on an integer scale from 0 (lowest) to 10 (highest). Individual‐specific stressor events were assessed by asking participants to indicate if any of the following changes in life circumstances had occurred (multiple‐choice questions): conflict or difficulties with spouse or partner (“Schwierigkeiten mit dem Ehepartner/in, Freundin/Freund,Lebensgefährten”), family‐related stress (due to children, parents or other relatives) (“Belastung durch Versorgung von Kindern (Homeschooling), Eltern oder anderen Angehörigen”), loss of social contacts/loneliness (“Verlust sozialer Kontakte, Einsamkeit”) (social stressors), work‐related stress (“Stress bei der Arbeit”), financial stress (“finanzielle Sorgen/Probleme”), housing issues/cramped living conditions (“Belastung durch beengte Wohnverhältnisse”) and unspecified other stress (note that the survey question explicitly asked for changes, even though some answer options could be interpreted as permanent states).

### 2.3 Statistical Procedure

Sample characteristics were calculated as absolute and relative frequencies for categorical variables and means with standard deviations for continuous variables. Associations among repeatedly measured variables from the app‐based survey (e.g., mental and physical health) were examined using repeated‐measures correlation as implemented in the *rmcorr* package in R. This method estimates a common within‐person correlation by fitting parallel regression lines with a shared slope, thereby controlling for stable between‐person differences (Bakdash and Marusich 2017). For comparison, between‐person correlations were computed by averaging each variable within participants and then calculating Pearson correlations among these person‐level means.

As motivated in the introduction, we fitted Bayesian mixed ordinal regression models. Each response variable (subjective mental health, physical health, fear, sadness, and anger) was modeled separately using a cumulative ordinal logistic regression with participant‐level random intercepts. The seven stressors included as fixed effects were: (1) conflict with spouse or partner, (2) family issues, (3) loss of social contacts, (4) work‐related stress, (5) financial issues, (6) housing issues, and (7) other stress. Furthermore, we adjusted for age, sex and employment (yes/no).

For participant i at time j, the model is defined as:

$$\begin{array}{ll} \text{logit}\left(P\left(Y_{i,j}\leq k\right)\right)=\theta_{k}- & \left(\beta_{1}\;{\text{ConflictSpouse}}_{i,j}+\beta_{2}\;{\text{FamilyStress}}_{i,j}+\beta_{3}\;{\text{Loneliness}}_{i,j}\right.\\ & +\beta_{4}\;{\text{WorkStress}}_{i,j}+\beta_{5}\;{\text{Financial}}_{i,j}\\ & +\beta_{6}\;{\text{Housing}}_{i,j}+\beta_{7}\;{\text{OtherStress}}_{i,j}\\ & \left.+\beta_{8}\;{\text{age}}_{i,j}+\beta_{9}\;{\text{sex}}_{i,j}+\beta_{10}\;{\text{employment}}_{i,j}+b_{i}\right) \end{array}$$

Where.

- $Y_{i,j}$ is the ordinal response (e.g., subjective mental health) for participant i at time j,
- $\theta_{k}$ are threshold (cutpoint) parameters for response category k,
- $\beta_{1},\text{…},\beta_{7}$ are fixed‐effect coefficients for the seven stressors,
- $b_{i}∼N\left(0,\sigma_{b}\right)$ is a participant‐specific random intercept.

The random intercepts estimate the psychological resilience of the participant during the study period because they explain the difference between the predicted value (of the dependent variables) and the stressor exposure of the individual (up to the random error). More precisely, the random intercept is the difference of the person‐specific intercept to the average intercept. With constant slopes, it is the difference between the person‐specific prediction and the average prediction conditional on a specific value of the stressor. So, for the same exposure, some participants report better subjective health as compared to the average, and we call that difference “resilience”. Note that the random intercept estimate (for any participant) depends on the entire sample, as a relative notion of resilience and that it is a stable, time‐invariant indication for the given time window of the study. As we estimate separate models for the various dependent variables, we get a resilience estimate in terms of each dependent variable. The estimates for the association between stressors and the dependent variables are presented as odds ratios with 95% credible intervals. As we re‐ordered the values of the dependent variable such that higher values are better, the ORs less than 1 indicate that higher stress is associated with worse outcomes, while ORs greater than 1 indicate the opposite.

**FIGURE 1:**
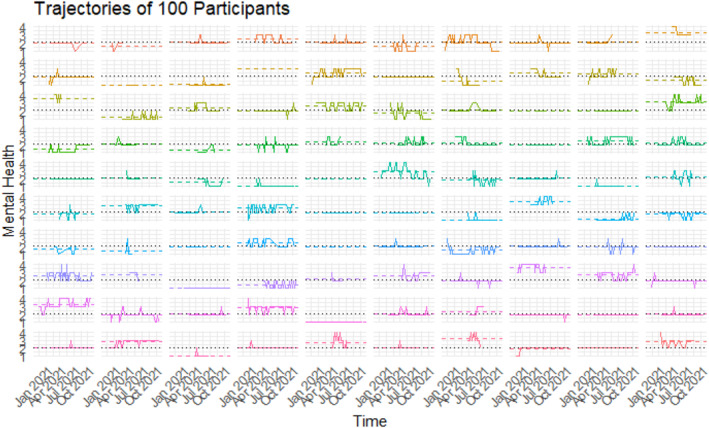
Individual trajectories of a random sample of 100 study participants. Black dashed lines for the population average, participant‐colored dashed lines for the participant’s average.

To determine whether random slopes were warranted in the model, we plotted individual response trajectories over time for a random subset of 100 participants, see Figure 1. These plots revealed that most participants' trajectories differed primarily in their baseline level (i.e., intercept), while the within‐person variability over time was minimal and often limited to transitions between two adjacent ordinal levels (e.g., from 1 to 2, or 2–3). This supported the use of a random intercept model to account for between‐subject heterogeneity in overall response level.

We further evaluated a random slopes model by introducing random effects (for participant id) with a relatively informative prior (N(0,0.1)) to encourage stable estimation. However, the model failed to converge after 2400 iterations, as indicated by $\hat{R}$ > 1.01 for key parameters. Despite this, a comparison using approximate leave‐one‐out cross‐validation (LOO) showed that the random intercept model yielded a better expected log predictive density (elpd), further supporting the more parsimonious specification.

Parameter estimation was based on MCMC sampling (from the posterior distribution) with 4 chains with 2000 iterations each, amounting to 4000 post‐warmup draws, with no thinning (thinning = 1) and non‐informative priors (default setting). The likelihood corresponds to a cumulative ordinal model with logit link function. Bulk and tail effective sample size measures exceeded 2700 for all parameter estimates. To assess convergence and posterior sampling quality, we followed current recommendations by inspecting trace plots, density overlays, and rank plots for all parameters and we checked $\hat{R}$ < 1.01 (Vehtari et al. 2021). The computations were performed by the statistical software *R* version 4.3.0 (R Core Team 2022). The Bayesian mixed ordinal regression models were fitted using the package brms (Bürkner 2018), which interfaces with the probabilistic programming language *Stan* (Team 2023).

## 3 Results

### 3.1 Sample Characteristics

The total number of app users was *N* = 7386, among them 3805 women (51.52%). The app users' mean age was 55.09 with a standard deviation of 13.62. In total, they submitted 206,912 responses to the app survey, among them 107,119 reports by women (51.77%). The average number of responses per participant was 28.01 with a standard deviation of 14.70. There were 86.4% (6380 out of 7380) participants who provided at least 10 responses each. The number of responses per participant had a median of 30 and an inter‐quartile range of 23, ranging from min 1 to max 257 responses. Descriptive sample statistics are shown in full in Table 1.

**TABLE 1:** Descriptive sample statistics.

	Available data (n)	Sample
Age (mean, SD)	7386	55.09 (13.62)
Sex (female, %)	7386	3805 (51.52%)
Employment	6629	
Employed		4393 (66.27%)
Unemployed		93 (1.4%)
Housewife/Houseman		162 (2.44%)
Parental leave		131 (1.98%)
In education/apprenticeship		133 (2.01%)
Retired		1326 (20%)
Mode of work	4859	
Exclusively at home		1204 (24.78%)
Mostly at home		941 (19.37%)
Mostly on‐site		833 (17.14%)
Exclusively on‐site		1881 (38.71%)
COVID tests and contacts (reported during app survey)	206,912	
Contact with confirmed case		2804 (1.35%)
Contact with suspected case		1507 (0.72%)
Being tested during previous 7 days		44,546 (21.58%)
Being currently under quarantine		1575 (0.76%)

In particular, 4393 out of 6629 (66.27%) were employed, while 1326 (20.00%) were retired. There were 2804 out of 206,912 responses (1.35%) of an encounter with a confirmed case and 1507 reports (0.72%) of an encounter with a suspected case. There were 1575 reports (0.76%) of currently being under quarantine.

### 3.2 Distribution and Associations of Dependent Variables and Stressors

Among all app survey responses, both physical and mental health were sharply concentrated at the mode “good” with frequencies 155,418 (75.11%) and 141,932 (68.60%) respectively. Across individuals (between‐person correlations), physical and mental health were strongly related (*r* = 0.76). Negative affective states also showed pronounced clustering, with especially high correlations between fear and sadness (*r* = 0.74), sadness and anger (*r* = 0.64), and fear and anger (*r* = 0.59). The pattern of within‐person (repeated‐measures) associations was similar but attenuated in magnitude. Again, mental and physical health showed the strongest coupling (*r* = 0.42). Negative affects were also strongly interconnected within persons, most notably fear–sadness (*r* = 0.34), sadness–anger (*r* = 0.31), and fear–anger (*r* = 0.31). Among the health–affect associations, (bad) mental health was most closely linked to sadness (*r* = 0.31).

The most common kind of stress was work‐related stress with 6614 out of 199,619 (3.31%) reports. Subsequently, stress due to loss of social contacts occurred in 3997 (2.00%) reports, stress with children or elderly relatives in 3909 (1.96%) reports, and stress with spouse or partner in 3439 (1.72%). The category of unspecified “other stress” was reported in 2543 cases (1.27%). Slightly less common were financial problems in 1940 (0.97%) reports and housing issues in 746 (0.37%) reports.

### 3.3 Results of Mixed Model Analysis: Impact of Daily Stressors

Across all outcomes, values were ordered s.t. higher values indicate better health or lower negative affect. Therefore, odds ratios (ORs) below 1 reflect worse outcomes with increasing stress, whereas ORs above 1 reflect better outcomes.

#### 3.3.1 Mental Health

**FIGURE 2:**
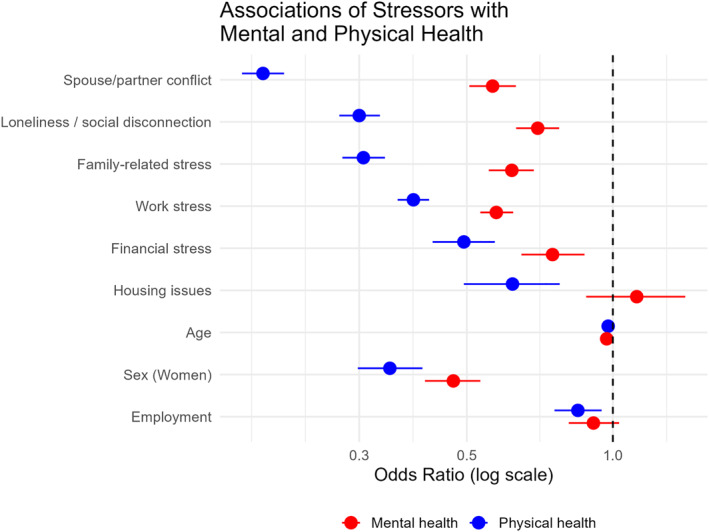
Odds ratios for associations between health (ordered such that higher values indicate better health) and daily stressors, with 95% CIs, obtained from fixed effect coefficients of ordinal mixed model with covariate adjustment by age, sex and employment.

All stressors showed significant negative associations with mental health (all ORs < 1), after adjusting for age, sex, and employment, see Figure 2. The strongest associations were observed for conflicts with spouse/partner (OR = 0.19, 95% CI [0.17, 0.21]), family‐related stress (OR = 0.31, 95% CI [0.28, 0.34]), and loss of social contacts/loneliness (OR = 0.30, 95% CI [0.27, 0.33]). Work‐related (OR = 0.39, 95% CI [0.36, 0.42]) and financial stress (OR = 0.49, 95% CI [0.43, 0.57]) were also associated with poorer mental health, though to a lesser extent. Housing issues showed the weakest, but still significant, association (OR = 0.62, 95% CI [0.49, 0.78]).

#### 3.3.2 Physical Health

Associations followed a similar pattern but were attenuated compared with mental health. The strongest associations were found for conflicts with spouse/partner (OR = 0.56, 95% CI [0.51, 0.63]), family‐related stress (OR = 0.62, 95% CI [0.56, 0.69]), and loneliness (OR = 0.70, 95% CI [0.63, 0.78]). In contrast to mental health, housing issues were not significantly associated with physical health (OR = 1.12, 95% CI [0.88, 1.41]).

#### 3.3.3 Negative Affect (Fear, Sadness, Anger)

**FIGURE 3:**
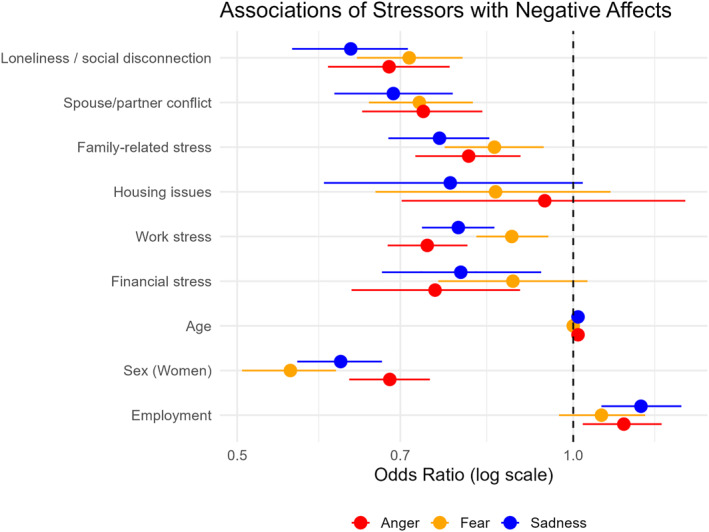
Odds ratios for associations between negative affects (ordered such that higher values indicate lower negative affect) and daily stressors, with 95% CIs, obtained from fixed effect coefficients of ordinal mixed model with covariate adjustment by age, sex and employment.

All stressors showed significant associations with negative affect (ORs < 1), except for housing issues and fear or sadness, see Figure 3.

For fear, the strongest associations were with loneliness (OR = 0.71, 95% CI [0.64, 0.80]) and conflicts with spouse/partner (OR = 0.73, 95% CI [0.66, 0.81]).

For sadness, the largest effects were conflicts with spouse/partner (OR = 0.69, 95% CI [0.61, 0.78]) and loneliness (OR = 0.63, 95% CI [0.56, 0.71]).

For anger, the strongest associations were conflicts with spouse/partner (OR = 0.73, 95% CI [0.65, 0.83]) and loneliness (OR = 0.68, 95% CI [0.60, 0.78]).

#### 3.3.4 Overall Pattern

Across all outcomes, interpersonal and social stressors (conflicts with spouse/partner, family stress, loneliness) consistently showed the largest associations, whereas work‐related, financial, and especially housing‐related stressors exhibited comparatively weaker effects.

### 3.4 Impact of the COVID‐19 Pandemic Over Time

**FIGURE 4:**
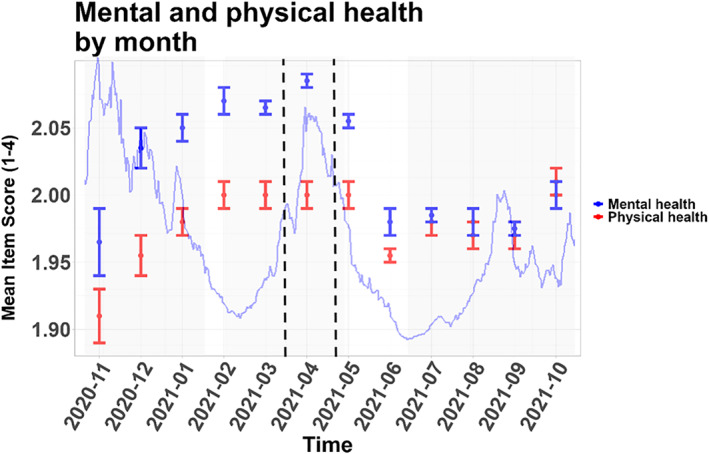
Monthly averages of subjective mental and physical health of the cohort, with confidence intervals. Incidence in Mainz (Germany) is shown with the blue line in the background. Vertical dashed lines indicate start and end of the lockdown in Mainz.

The average subjective physical health was worst from February to May 2021 and in October 2021, while the average subjective mental health was worst from January to May 2021, see Figure 4.

**FIGURE 5:**
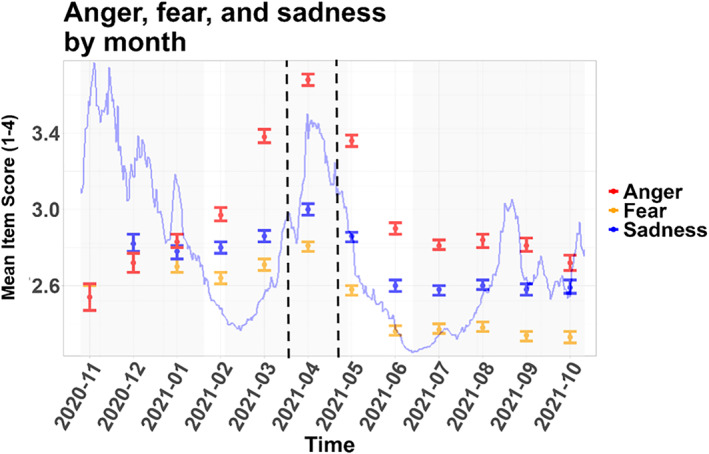
Monthly averages of fear, sadness and anger of the cohort, with confidence intervals. Incidence in Mainz (Germany) is shown with the blue line in the background. Vertical dashed lines indicate start and end of the lockdown in Mainz.

Fear had a declining trend with local peaks in December 2020 and in April 2021 (for this and the following see Figure 5). Sadness had elevated levels from December 2020 to May 2021, with a peak in April 2021. Anger had a peak in April 2021, with also elevated values in the adjacent months of March and May 2021.

During April 2021 a lockdown was implemented in Mainz and Mainz‐Bingen (and later nationally). This concerns calendar weeks 13–19 of 2021, where calendar week 13 lasted from March 29 to April 4, 2021 and calendar week 19 lasted from May 10 to May 16, 2021.

## 4 Discussion

Both mental and physical health were most strongly associated with social stressors: conflicts with spouse or partner, family‐related stress, loss of social contacts and loneliness. These stressors had a stronger association with health and well‐being than work‐related stress, despite the latter being reported most frequently. The heightened sensitivity to these interpersonal and social stressors is consistent with findings from other studies, which highlight the critical role of social support and relationships in maintaining mental well‐being (Umberson and Karas Montez 2010). Therefore our mixed‐model‐based finding that social stressors had the strongest associations with subjective health are very plausible given the changed social conditions during the pandemic: for instance, during the pandemic parents and their adolescent children suffered from a significant increase in psychosocial stress, which was even significantly higher amongst mothers, possibly due to care responsibilities and a generally higher vulnerability to stress disorders (Connor 2020; Paschke et al. 2021; Petersen et al. 2022). Furthermore, it has been found that financial problems or cramped living conditions can lead to social relationship problems, that is that social relationship issues could act as a mediator for worse psychological outcomes (Grant 2003).

A sizable portion of the stressor “loss of social contacts” could maybe be attributed to the COVID‐19 pandemic and the associated contact restrictions. The pandemic has exacerbated feelings of isolation and loneliness, which have been linked to poorer mental health outcomes (Loades et al. 2020).

Our analysis of the GCS cohort study indicates that subjective mental health is more strongly associated with stressors than subjective physical health. Furthermore, there was a strong correlation between mental and physical health, which could be explained by previous findings such as that physiological disease is often preceded by psychological stress (Cohen et al. 2007).

Our resilience approach, using Bayesian mixed models for ordinal data, applied to the Gutenberg‐COVID‐19 study, therefore gave very plausible estimates and results for various stressors. For further validation of the approach, it would be desirable to confirm significant associations of the random intercepts (resilience estimates) with potential risk factors (as found in the literature with previous methods).

The monthly averages of the dependent variables suggest short‐term negative effects of the pandemic (e.g., in terms of the peak of anger and fear in April 2021 during the April 2021 lockdown) and yet, a certain degree of resilience toward the pandemic in the long‐term (in terms of the return of the values of the dependent variables to previous values.) The short‐term negative effect is very plausible given the variety of pandemic‐related stressors, such as health anxiety, economic uncertainty, and the general disruption of daily life (Pfefferbaum and North 2020). Although the long‐term recovery of the population has been noted before (Pfefferbaum and North 2020), it would be interesting to find a more precise (ideally causal) quantification of the pandemic as an overall stressor (and the resilience to it).

A recent Cochrane review established very‐low certainty evidence that resilience of healthcare professionals can be strengthened directly through targeted psychological interventions (Angela et al. 2020). Although our study did not assess intervention effects, acknowledging this literature highlights that resilience is not solely reactive to stressors but can also be actively developed, which has implications for future research and preventive approaches.

### 4.1 Strengths and Limitations

A key strength of this study is the large sample size, and the relatively frequent longitudinal data collection of the app survey, which allowed mixed modeling. Note however, that as mentioned in the method's section, the number of responses (and the time points when they were submitted) varied among the participants.

The subjective physical and mental health, as well as affects, are self‐assessed and self‐reported (in particular, neither an official diagnosis nor measured on a validated scale). It would be interesting to consider objective assessments of the dependent variables (where available) which we could not do here as our data did not include any objective measures of mental and physical health.

As the app assessment did not include a rating of the severity of a given stressor (which is often part of the conceptual psychological definition of stress or stressor), we could not include individual stressor severity in our analysis.

Another limitation is that our analysis did not consider changes in resilience over time, which would require the addition of time × stressor interaction terms in the mixed models (and a longer study time window).

In this study, we treated individual daily stressors (such as work‐related stress) and the pandemic overall as independent stressors and we also did not include any interaction terms between different individual daily stressors, because while in reality there might be some complex interaction between them, there were no significant correlations between the daily stressor variables in our data and the quantification of the pandemic stressor would have been very ambiguous and debatable.

## 5 Conclusion

Overall, our findings highlight the critical potential of addressing social and interpersonal stressors to protect both mental and physical health. Interventions aimed at enhancing social support, reducing conflicts in close relationships, and mitigating feelings of loneliness could be valuable topics for further investigations into their beneficial effect. The monthly sample means of the dependent variables of the entire cohort suggest that while the pandemic has posed significant challenges, there is also a capacity for adaptation and recovery.

Further research is needed to gain a deeper understanding of how resilience is shaped by the interaction between social stressors and social support, and to more precisely and causally, quantify the role of the pandemic.

## Author Contributions

**Markus Schepers:** conceptualization, formal analysis, investigation, methodology, software, visualization, writing – original draft, writing – review and editing. **Irene Schmidtmann:** validation, writing – review and editing. **Sarah K. Schäfer:** methodology, validation, writing – review and editing. **Simge Yilmaz:** writing – review and editing. **Rieke Baumkötter:** writing – review and editing. **Alica Hartmann:** writing – review and editing. **Julia Petersen:** validation, writing – review and editing. **Nora Hettich‐Damm:** writing – review and editing. **Philipp Wild:** funding acquisition, project administration. **Daniela Zahn:** investigation, methodology, supervision, writing – review and editing. **Daniel Wollschläger:** investigation, methodology, supervision, writing – review and editing.

## Funding

The study was funded by the European Regional Development Fund and the Ministry of Science and Health of the State of Rhineland‐Palatinate (EFRE/REACT‐EU, Grant Nos. 84007232 and 84009735); by the ReALity Initiative of the Life Sciences of the Johannes Gutenberg University Mainz for the establishment of a cell bank; and by the National University Medicine Research Network on Covid‐19 (“NaFoUniMedCovid19”, Grant No. 01KX2021) B‐FAST for the topics “poor living conditions” and “working conditions” and their association with COVID‐19 in the population. Biomaterials were stored at the BioBank Mainz of the University Medical Center Mainz.

## Ethics Statement

The studies involving human participants were reviewed and approved by the Ethics Committee of the Rhineland‐Palatinate Medical Association as well as the Data Protection Officer of the Johannes Gutenberg‐University Medical Center Mainz. The patients/participants provided their written informed consent to participate in this study.

## Conflicts of Interest

The authors declare no conflicts of interest.

## Data Availability

Written consent from GCS study participants does not allow public access to the data. Access to the data in the local database is possible at any time upon request according to the ethics vote. This concept was developed with the local data protection officer and the ethics committee (local ethics committee of the Rhineland‐Palatinate Medical Association, German). Interested scientists can make their requests to the Gutenberg COVID‐19 Study Steering Committee (E‐Mail: info-gcs@unimedizin-mainz.de).
